# WWAD: the most comprehensive small molecule World Wide Approved Drug database of therapeutics

**DOI:** 10.3389/fphar.2024.1473279

**Published:** 2024-09-18

**Authors:** Polina Savosina, Dmitry Druzhilovskiy, Dmitry Filimonov, Vladimir Poroikov

**Affiliations:** Laboratory of Structure-Function Based Drug Design, Department of Bioinformatics, Institute of Biomedical Chemistry, Moscow, Russia

**Keywords:** approved drugs database, cheminformatics, computer-aided drug design, unified resource, World Wide Approved Drugs (WWAD)

## 1 Introduction

Every year, more than 50 new therapeutic agents are approved for medical use worldwide. Despite the increase in the number of biologics approvals, the majority of drugs currently available are chemical entities (de la Torre and Albericio, 2024). The development of small-molecule drugs continues to be favored due to the lower cost of research (Wouters et al., 2020), the wider range of applications, and availability to patients (Makurvet, 2021). However, drug development is a long-term process taking on average 10–15 years, and the cost of bringing a drug to market can be $2.5 billion (Catacutan et al., 2024).

The wide application of computer-aided drug design (CADD) methods allowed for a reduction in time and financial costs in the drug discovery process (Athanasiou and Cournia, 2019). Knowledge of existing therapeutics, including their structure, biological activity, physicochemical parameters, etc., is a necessary part of the development and application of CADD methods. Currently, numerous freely accessible curated chemical databases (DBs) containing information about approved drugs are available via the World Wide Web (Masoudi-Sobhanzadeh et al., 2020; Elkashlan et al., 2023).

Most of these resources contain data only on medicines approved in the US and Europe. However, up to 30% of medicines are approved for the first time by authorities in other countries (Wu et al., 2021), and this information is often missing from the existing DBs. In July 2024, for example, one could not find records in the widely used resource DrugBank (https://go.drugbank.com) for the anticoronavirus drugs simnotrelvir and leritrelvir, which were approved in China in 2023 (Zhu, 2023; Zheng et al., 2024), despite their relevance to the therapy of COVID-19.

The lack of locally authorized drug information in freely available resources does not allow researchers to use this knowledge in the development and application of CADD methods. It significantly constricts the investigated pharmacotherapeutic chemical space and reduces the known structure-activity relationships. Thus, there is an urgent need to create an open-access database containing information on medicines, irrespective of the country of their origin and use.

The National Medicine Registers (NMRs) are the most reliable sources of data on pharmaceuticals authorized for therapeutic use. Despite the free availability of NMRs, a search for them is challenging due to the large number of regulatory authorities in the world (World Health Organization, 2024) and the lack of a unified resource providing the appropriate access (Siramshetty et al., 2022). The variety of report presentations and document formats published by regulatory authorities also complicates the collection and aggregation of information on the approved medicines, since it is not possible to apply a uniform processing algorithm to retrieve such data.

Taking into account the needs for a single source of aggregated information on worldwide approved medicines, we created the World Wide Approved Drugs (WWAD) database. WWAD contains information collected manually from the NMRs and official documentation from the medicines regulatory authorities (MRAs) of different countries, which are freely available on the Internet.

## 2 Materials and methods

**FIGURE 1:**
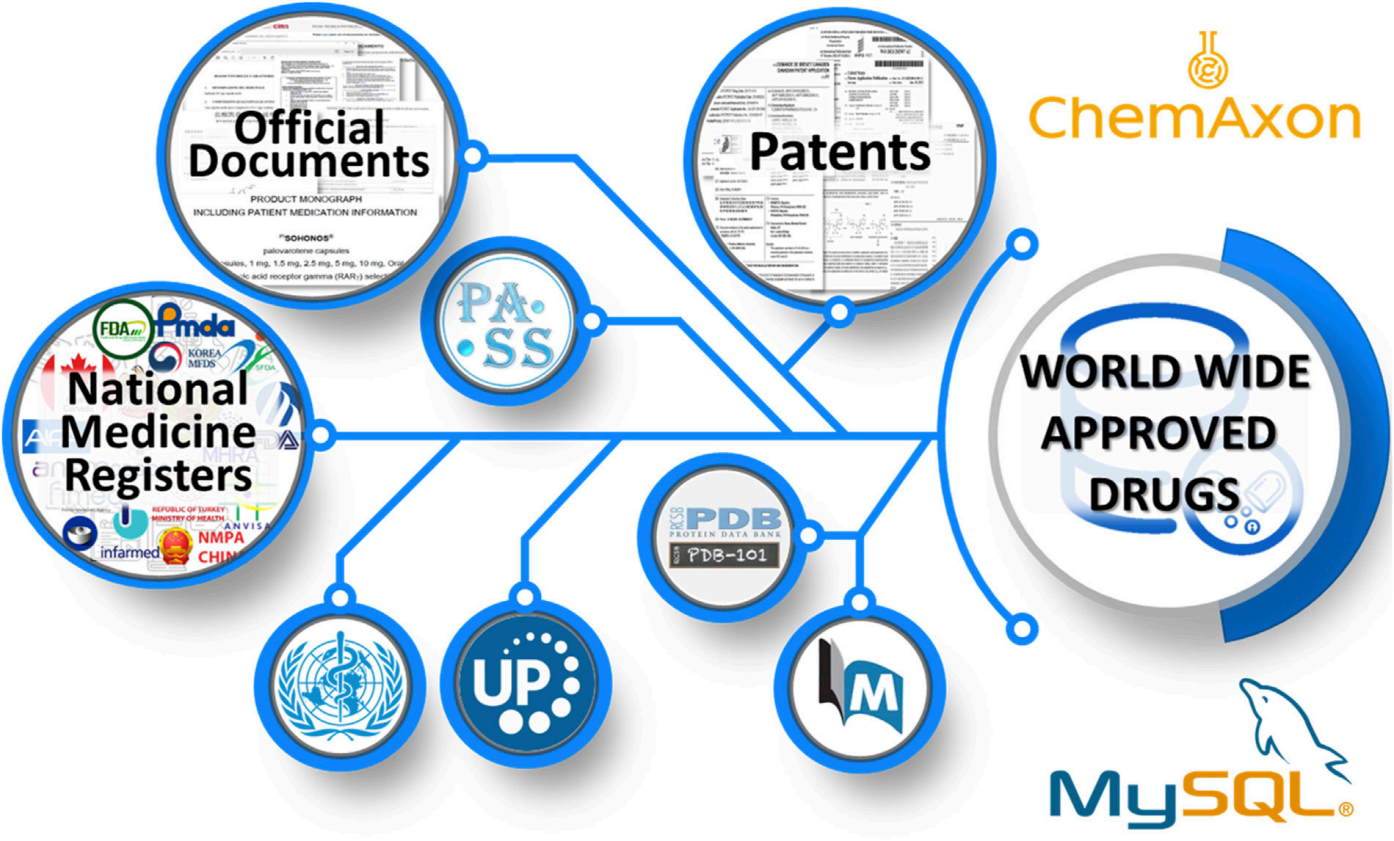
The WWAD database creation scheme.

The information collection and handling process for the WWAD database consisted of eight stages (Figure 1). The relational database management system MySQL and the program suite Instant JChem (IJC) from ChemAxon (ChemAxon, 2024a) were used for storing and processing the collected data.

First of all, we composed a preliminary set of pharmaceutical substances presented in the existing DBs. Next, we searched the NMRs of the MRAs of different countries, which are freely available on the Internet. Currently, we have found the freely available pharmaceutical registers of 71 countries and regions: Argentina, Armenia, Australia, Austria, Bahrain, Belarus, Belgium, Bhutan, Bosnia and Herzegovina, Brazil, Bulgaria, Cameroon, Canada, China, Croatia, Czech Republic, Denmark, Egypt, Estonia, European Union, Finland, France, Germany, Greece, Hong Kong, Hungary, Iceland, India, Ireland, Israel, Italy, Japan, Kazakhstan, Republic of Korea, Latvia, Lebanon, Liberia, Lithuania, Malaysia, Malta, Mexico, Netherlands, New Zealand, Nigeria, Norway, Pakistan, Philippines, Poland, Portugal, Romania, Russia, Saudi Arabia, Serbia, Singapore, Slovakia, South Africa, Spain, Sweden, Switzerland, Tanzania, Thailand, Turkey, United Arab Emirates, Uganda, Ukraine, United Kingdom, United States, Uruguay, Uzbekistan, Zambia, and Zimbabwe. The preliminary set of low molecular weight pharmaceutical substances approved for medical use was significantly expanded using this data.

The lists of approved drug names were extracted from the identified NMRs. Then, the names of vaccines, biologics, traditional medicines, and homeopathic products were deleted from the collected lists because the information about such products is not used in most CADD methods (Fourches et al., 2010; Mansouri et al., 2024).

Further, using the MedNet information service provided by the World Health Organization (INN-Mednet, 2024), the drug names from the collected lists were matched with their international non-proprietary names (INN) in seven languages (English, Russian, French, Spanish, Latin, Arabic, and Chinese) and national non-proprietary names (for example, the United States adopted name and British approved name). Thus, we created a unified dictionary of the therapeutics, where drug names were presented by their INN in English. If INN was not available for a compound, we used the shortest wide-spread name: a well-established (e.g., morphine, codeine), national (e.g., carotegrast methyl), trivial (e.g., acetic acid), or trade name (e.g., dimolegin), etc. The Anatomical Therapeutic Chemical (ATC) classification system codes of the substances were also obtained using the MedNet service.

At the next stage, a search for official documents published by the MRAs after the drug approval process was conducted. A set of official documents that were retrieved for the WWAD creation includes a Summary of Product Characteristics, FDA Drug Label, Drug Monograph, Instructions for Use, European Public Assessment Report, Pharmaceuticals and Medical Devices Agency Review Report, and FDA Pharmacology Review. The last three documents are the most informative and contain the results of the comprehensive review of the drug, which is necessary for the issuance of approval for medical use. Due to insufficient accuracy of the current text mining algorithms (Islamaj Dogan et al., 2019; Bachman et al., 2023; Leaman et al., 2023), all documents were processed and analyzed manually.

We composed the following fields for each compound: 1) a brief description of pharmacotherapeutic use, 2) a list of routes of administration, 3) information about molecular targets with interaction type, 4) therapeutic indications, and 5) biological activities. The molecular target names were unified using the UniProt database (UniProt Consortium, 2023). The biological activity terms were described based on the training set of the PASS (Prediction of Activity Spectra for Substance) software containing more than 8,000 activity terms (Filimonov et al., 2014; Filimonov et al., 2018). The list of standardized terms can be found on the web resource page (https://www.way2drug.com/wwad/terms.php). The ligand identifiers from the Protein Data Bank (Burley et al., 2023) were added to associate therapeutic agents with the known three-dimensional complexes of their targets. To expand the knowledge about interactions of a compound with molecular targets and their pharmacological activities, an additional search for information regarding the collected drugs was conducted in the PubMed DB (https://pubmed.ncbi.nlm.nih.gov).

The validation of chemical structures was performed based on the patent information searched via freely available web services: Espacenet (https://worldwide.espacenet.com), Lens (https://www.lens.org), Google Patents (https://patents.google.com), Yandex.Patents (https://yandex.ru/patents), and PATENTSCOPE (https://patentscope.wipo.int). The processing, standardization, and recording of structural formulas in the database were performed using the Marvin Sketch and Standardizer tools implemented in the IJC complex (ChemAxon, 2024c; ChemAxon, 2024d). The salts and solvents were removed from the compound structures, and the charged atoms were neutralized according to the currently adopted standards for chemical structure representation in CADD methods. Biologic, metal-organic, and inorganic compounds were filtered out because it is recommended to remove such entities from the training and test sets in the application of CADD methods (Fourches et al., 2010; Mansouri et al., 2024). The string structure representation and structure images were generated using the “MolString” and “MolImage” functions (ChemAxon, 2024b) from the IJC program complex. As a result, only low molecular weight organic compounds and human peptide analogs consisting of no more than 50 amino acid residues used for the treatment, prevention, and diagnosis of various diseases were added to the created DB, since such substances are considered non-biological drugs according to the current FDA guidelines (Otvos and Wade, 2023).

At the last step, prediction of biological activity spectra for the collected drug structures was carried out using the 2022 version of PASS software. The structure-activity relationship analysis is based on the Bayesian approach and has been described in detail in earlier publications (Filimonov et al., 2014; Filimonov et al., 2018). The PASS prediction results presented as a list of activities with the corresponding estimated probabilities “to be active” (Pa) and “to be inactive” (Pi) that fulfilled Pa > Pi criteria were also stored in the database.

## 3 World Wide Approved Drugs database

### 3.1 Database structure

Based on the information presented in the NMRs, official documents, and the minimal set of properties that are necessary for the CADD method application, we defined the following fields for each pharmaceutical substance:

• Name and synonyms.
• Chemical structure in MOL, SMILES, and InChI formats and its image in PNG format.
• ATC classification system codes.
• The approval information in the country or region with the name of the MRA, approval date, therapeutic indication, and a link to the data source.
• Set of routes of administration.
• Name of the active substance that realizes a direct interaction with therapeutic molecular targets or the main pharmacotherapeutic effect.
• List of molecular targets with an indication of the type of interaction and a corresponding link to the data source.
• Set of therapeutic indications with a link to the data source for each item.
• List of the possessed biological activities with a corresponding data source link.
• Result of the prediction of biological activity spectra performed by PASS software.

For prodrugs, the following approach to describing molecular target interactions was implemented: if an active moiety was approved as an independent therapeutic agent, only targets with which a prodrug interacts were presented in the set of molecular targets, and the relationship with the WWAD record for the corresponding active substance was realized through the appropriate field; if the information about the approval of an active moiety as an independent drug was not found, all molecular targets were presented with the name of the interacting substance in the field.

### 3.2 The current version of the WWAD database

**FIGURE 2:**
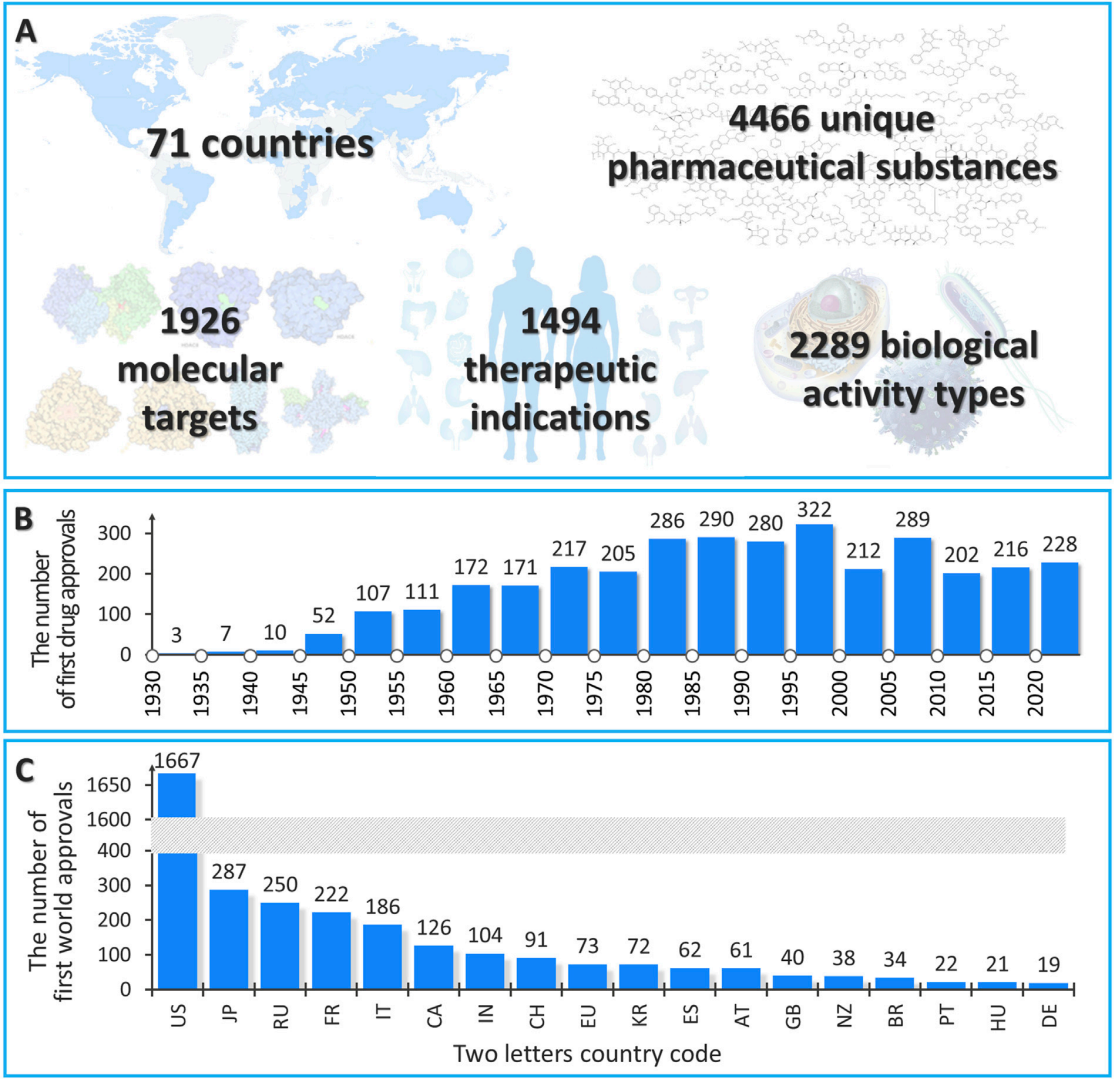
General statistics of the current version of the WWAD database. **(A)** The number of countries, drug substances, molecular targets, therapeutic indications, and biological activity types in the current version of the WWAD database. **(B)** The number of drugs first approved from 1930 to 2023 based on the WWAD data. **(C)** The number of drug substances approved in certain countries, NMRs of which were analyzed, for the first time in the world (the countries in which more than 10 drugs were authorized for the first time in the world are presented).

Information about the WWAD current version is presented in Figure 2A. At present, the WWAD database contains information on 4,466 unique pharmaceutical substances, of which 3,504 drugs were approved between 1930 and 2024 (Figure 2B). For the other 962 drugs, information about the dates of approval was not available. It should be noted that a large number of drugs with the first approval from 1980 to 2000 may be due to the fact that the majority of NMRs do not contain any earlier information on drug approvals. Based on the collected data, we noticed that a number of drugs that got their initial approval in the United States (1,667 drugs) is comparable to the cumulative number of drugs approved for the first time in other countries (1,837 drugs) (Figure 2C). This supports the need to collect information from the NMRs of different countries and regions for the most comprehensive analysis of the known pharmacotherapeutic chemical space.

The total number of molecular targets with which drugs in the WWAD interact is 1,926 biomacromolecules. The largest number of substances have histamine receptors as the main therapeutic target (more than 90 drugs that are histamine receptor antagonists). In terms of transporter interactions, the majority of drugs are P-glycoprotein substrates, inhibitors, or inducers (more than 500 drugs). Cytochrome P450 3A4 is the metabolic enzyme for the majority of compounds in the WWAD (more than 500 substances). The total number of diseases for which the drugs presented in the WWAD are prescribed as remedies for treatment, prevention, or diagnosis makes 1,494. The most common therapeutic indication is a bacterial infection (for more than 280 drugs). In total, we identified 2,289 different biological activities exhibited by the collected drugs. The majority of the WWAD substances possess anti-infective activity (more than 240 drugs).

We have implemented a web resource on the Way2Drug platform (https://www.way2drug.com/wwad/) to make it freely available for academic use in the Scientific Community. For security, registration is required for each user. All registered users have access to the information about 3,776 pharmaceutical substances not related to narcotic, psychotropic, and toxic compounds. According to the current laws of the Russian Federation and some other countries, open access to such data is prohibited. A structural formula, a brief description of the pharmacotherapeutic application, sets of molecular targets, indications and other types of biological activity, information about the first approval for clinical use, PDB ligand identifiers, and PASS prediction results are specified for compounds presented at the web resource.

The WWAD data are presented in an interactive table, which allows either a global search or filtering of data presented by a drug name, therapeutic indication, short name of the regulatory authority that granted the first approval, and the first approval date. Information on molecular target interactions and approvals for therapeutic use is enhanced by the data source links.

## 4 Possible applications

Information from the WWAD database can be used in the biomedical area for various purposes, including but not limited to the knowledge discovery, drug repositioning, *in silico* methods benchmarking, and novelty assessment of the known and newly investigated drug-like compounds.

### 4.1 Searching information about the approved drugs

WWAD may be used for search of information on the approved drugs, including target interactions, therapeutic indications, etc. For example, we used WWAD to analyze pharmacotherapeutic groups of drugs that were investigated as anticoronavirus agents in high-throughput *in vitro* screens (Savosina et al., 2021).

### 4.2 Drug repositioning

The structures of pharmaceutical substances assembled at WWAD can be used to predict the biological activity for drug repositioning. For example, a virtual screening using molecular docking, molecular dynamics modeling, and binding energy estimation was performed to search for inhibitors of the main protease of the SARS-CoV-2 virus in the WWAD chemical space (Skvortsov et al., 2020). The results of PASS biological activity spectra prediction for collected compounds available through the developed web resource can also be used in drug repurposing. For instance, a wide range of antineoplastic effects is predicted for the antimalarial pyronaridine. The experimental evidence confirming this prediction was found in the literature: pyronaridine exerts cytotoxicity on human breast cancer and leukemia cell lines (Villanueva et al., 2018). A clinical trial has also been announced to study pyronaridine for treatment of acute lymphoblastic leukemia and myeloid leukemia (ClinicalTrials.gov, 2022).

### 4.3 Data enrichment

Most widely used web resources contain information on a limited number of drugs, mainly only those approved in the United States and Europe. It should be kept in mind that the overall relative drug gap in emerging markets has now narrowed significantly and that the disparity in drug availability is largely driven by genetic, phenotypic, and, not unreasonably, political economy motives. With this in mind, reliable information on therapeutic agents developed and approved locally in one or two countries, for example, on the basis of ethnicity peculiarities, remains unknown (Maglo et al., 2016). It is also worth considering that drugs developed in one country are significantly delayed by regulatory authorities in other countries due to the economic inexpediency (Wileman and Mishra, 2010; Mulcahy, 2024). In particular, the cholinesterase inhibitor neostigmine has been approved in Russia since 1964, but was approved in the United States only in 2013. Another example is the atypical antipsychotic amisulpride, which was first approved in France on 20 January 1986, but was not approved by the FDA until 2020. All this deprives researchers of native information about already known drug-like compounds, and moreover, those used in clinical practice, including the studied effects, mechanisms of action, side and toxic effects, and drug-drug interactions, which may determine the quality of the appropriate (Q)SAR models.

### 4.4 Novelty of the investigated compounds by similarity assessment

Since WWAD contains information on drugs approved in different countries and regions, our web resource can be used to evaluate the novelty of the investigated molecules. For example, in the recent study, we estimated a structure similarity between the WWAD substances and the novel hybrid dithioloquinolinethione derivatives, which were studied as anti-inflammatory agents. The novelty of these compounds was shown not only in the chemical space of anti-inflammatory compounds but also among the drugs from the other pharmacotherapeutic areas. Also, we used WWAD to determine the cutoffs for some structural similarity assessment methods. For this purpose, pairwise comparisons of compound structures were analysed both for all molecules and for drugs of the same pharmacotherapeutic group, followed by the frequency analysis (Medvedeva et al., 2024).

## 5 Conclusions

The information about the approved low molecular weight drug substances is necessary for the application of CADD methods, which are widely used in pharmaceutical research and development. However, the knowledge presented in the existing drugs DBs is insufficient because they highlight the United States and EU medicine’s regulatory authority data only. Local therapeutics approved outside these countries are not included, which significantly constricts the investigated pharmacotherapeutic chemical space.

We created a unique World Wide Approved Drugs database, where information about low molecular weight pharmaceutical substances from the NMRs of 71 countries and regions is aggregated. A standardized representation of structural formulas and terms describing biological activity allows the WWAD data to be used in CADD methods without preprocessing. The use of official documents published by the MRAs after the approval process as the main data source provides the veracity of the information collected. The freely available for academic purposes web resource allows access to the aggregated data for a wide range of researchers.

## Data Availability

The original contributions presented in the study are included in the article. WWAD is openly available at https://way2drug.com/wwad. Further inquiries can be directed to the corresponding author.

## Author Disclaimer

The WWAD database copyrights have been registered by Rospatent: No. 2024621221, 21.03.2024.

## Abbreviations

ATC, anatomical therapeutic chemical; CADD, computer-aided drug design; DB, database; IJC, Instant JChem; INN, international non-proprietary name; MRA, medicines regulatory authority; NMR, national medicine register; WWAD, World Wide Approved Drugs.
